# Taming the Reactivity of Monoterpene Synthases To Guide Regioselective Product Hydroxylation

**DOI:** 10.1002/cbic.201900672

**Published:** 2019-12-03

**Authors:** Nicole G. H. Leferink, Kara E. Ranaghan, Jaime Battye, Linus O. Johannissen, Sam Hay, Marc W. van der Kamp, Adrian J. Mulholland, Nigel S. Scrutton

**Affiliations:** ^1^ Manchester Institute of Biotechnology and School of Chemistry University of Manchester 131 Princess Street Manchester M1 7DN UK; ^2^ Centre for Computational Chemistry School of Chemistry, University of Bristol Cantock's Close Bristol BS8 1TS UK; ^3^ School of Biochemistry University of Bristol University Walk Bristol BS8 1TD UK

**Keywords:** enzyme catalysis, molecular dynamics, protein engineering, synthetic biology, terpenoids

## Abstract

Monoterpenoids are industrially important natural products with applications in the flavours, fragrances, fuels and pharmaceutical industries. Most monoterpenoids are produced by plants, but recently two bacterial monoterpene synthases have been identified, including a cineole synthase (bCinS). Unlike plant cineole synthases, bCinS is capable of producing nearly pure cineole from geranyl diphosphate in a complex cyclisation cascade that is tightly controlled. Here we have used a multidisciplinary approach to show that Asn305 controls water attack on the α‐terpinyl cation and subsequent cyclisation and deprotonation of the α‐terpineol intermediate, key steps in the cyclisation cascade which direct product formation towards cineole. Mutation of Asn305 results in variants that no longer produce α‐terpineol or cineole. Molecular dynamics simulations revealed that water coordination is disrupted in all variants tested. Quantum mechanics calculations indicate that Asn305 is most likely a (transient) proton acceptor for the final deprotonation step. Our synergistic approach gives unique insight into how a single residue, Asn305, tames the promiscuous chemistry of monoterpene synthase cyclisation cascades. It does this by tightly controlling the final steps in cineole formation catalysed by bCinS to form a single hydroxylated monoterpene product.

## Graphical abstract

**Promiscuous chemistry of cyclisation cascades**: A synergistic experimental and computational approach revealed that Asn305 tightly controls the water attack and subsequent cyclisation step in the reaction cascade of bacterial cineole synthase, resulting in 95 % pure cineole via the (*S*)‐(−)‐α‐terpineol intermediate.

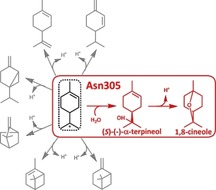

## Introduction

Monoterpenoids are industrially important natural products.^1^ Cineole (1,8‐cineole; eucalyptol) is used in the flavour, fragrance, and cosmetics industries due to its pleasant minty aroma and cooling spicy taste, as well as jet‐fuel precursor^2^ and sustainable solvent for organic transformations.^3^ Most known monoterpenoids, including cineole, are commonly produced by plants, where they play diverse roles in signalling and communication as well as defence against predatory species.^4^ As a result, most known monoterpene cyclases/synthases (mTC/Ss) are of plant origin. Recently, two mTC/Ss have been identified from the soil bacterium *Streptomyces clavuligerus*: a linalool/nerolidol synthase (bLinS), and a 1,8‐cineole synthase (bCinS).^5^

The modularity of terpene biosynthesis has been discussed previously,^6^ where plant mTC/S enzymes are bi‐domain enzymes comprising a C‐terminal class I terpene cyclase domain and a small N‐terminal domain of unknown function, bacterial mTC/S, consist of a single class I terpene cyclase domain only which is structurally related to bacterial sesquiterpene synthases.^7^ All mTC/S catalysed reactions involve unstable carbocation inter‐mediates which are shaped into a variety of linear and cyclic products along multiple reaction channels by the protein template before the reaction is terminated by deprotonation or nucleophilic attack.^8^ Both plant and bacterial class I terpene cyclase enzymes share two conserved metal binding motifs (the DDXXD motif and the DTE/NSE motif), which are involved in substrate binding and metal‐assisted ionisation of the geranyl diphosphate (GPP) substrate.^7^, ^9^ In addition, an effector triad, including a PPi sensor (Arg), linker (Asp), and effector (Gly) involved in ionisation, is strictly conserved in all class I terpene cyclases.^10^ After ionisation and subsequent formation of the first carbocation intermediate, the enzyme provides little more than a productive template for the cyclisation cascade. To prevent enzyme inactivation via active site alkylation, mTC/S enzymes possess relatively inert active sites, consisting of mainly polar and hydrophobic residues.^11^ As a result, there is little to no correlation between the sequence of amino acid residues associated with the active site and the cyclisation reaction catalysed. Plant enzymes have shown a high degree of functional plasticity, where a small number of mutations can drive changes resulting in rapid product diversification.^12^ As a result, many plant mTC/S enzymes produce monoterpenoid mixtures rather than a single clean product.^13^ For example, cineole synthases from *Salvia fruticosa*, *Citrus unshiu*, and *Arabidopsis thaliana* produce 65 %, 63 %, and 42 % pure cineole, respectively, and common by‐products include α‐terpineol, β‐pinene, sabinene and myrcene.^13^ Bacterial mTC/Ss have been shown to produce much cleaner product profiles. bCinS, for example, produces >95 % pure cineole from GPP with only very small amounts of by‐products.5a

**Figure 1:**
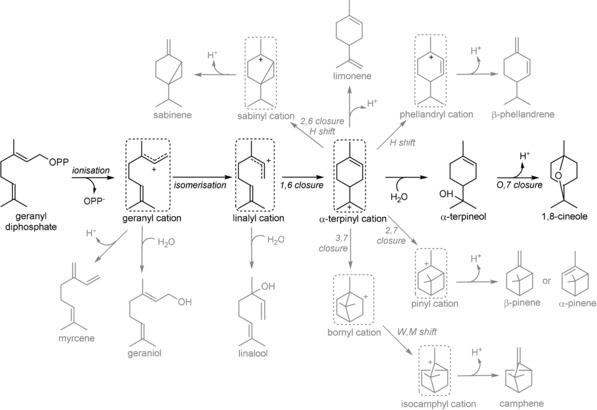
Proposed reaction cascade catalysed by CinS. Carbocation intermediates are shown in dashed boxes. The reaction starts with the metal‐dependent ionisation of geranyl diphosphate (GPP), resulting in the geranyl cation, which can undergo a range of cyclisations and hydride shifts before the reaction is terminated by deprotonation or nucleophilic attack. Common by‐products are shown in grey.

The proposed reaction cascade catalysed by cineole synthases is relatively complex comprising multiple steps, which provide several branching points and opportunities for premature quenching (see Figure 1). The reaction is initiated by a metal‐dependent ionisation of GPP resulting in the geranyl cation, the first carbocation intermediate. The geranyl cation subsequently isomerises to the linalyl cation (via linalyl diphosphate), which cyclises to form the α‐terpinyl cation, the first cyclic intermediate. The linalyl diphosphate intermediate is the first stereochemical intermediate, and determines the route via either (*S*)‐(−)‐ or (*R*)‐(+)‐α‐terpinyl, even though the final product cineole is achiral. The α‐terpinyl cation then undergoes nucleophilic attack by water at C7 to form either the (*S*)‐(−)‐ or (*R*)‐(+)‐α‐terpineol intermediate which undergoes proton induced cyclisation of the C=C double bond and the tertiary hydroxy group to form the final product 1,8‐cineole.^14^ Cineole synthase from *S. fruticosa* (CinS_Sf) is a well‐studied plant CinS: the crystal structure is known, and Asn338 was found to be essential in the reaction with water and formation of the α‐terpineol intermediate.

Removal of Asn results in no or drastically reduced α‐terpineol and cineole formation.12b CinS_Sf has also shown a high degree of functional plasticity, where a local deformation in a helix lining the active site is a major contributor to product specificity, and just a few mutations can convert CinS_Sf to a sabinene synthase.12b Unlike plant CinSs, bCinS is a unique mTC/S as it shows high fidelity and is thus capable of tightly controlling the carbocation intermediates during the cyclisation cascade towards the bicyclic product 1,8‐cineole with little to no branching and premature quenching. The crystal structure of bCinS has been solved previously,^7^ and Asn305 was proposed to be involved in stabilisation of a water molecule involved in water attack for the formation of the critical intermediate α‐terpineol, similar to Asn338 in CinS_Sf, however, these residues are located on opposite sides of the active site. Here, we investigate the role of Asn305 in the cyclisation cascade of bCinS using a synergistic approach combining experimental and computational methods. Our combined results give unique insight into how a single residue tightly controls cineole formation in bCinS and thereby prevents alternative products from accumulating.

## Results and Discussion

We used our previously established “plug‐and‐play” in vivo monoterpenoid production platform^13^ to rapidly determine product profiles of native and variant CinS enzymes without the need for protein purification. The platform consists of an engineered *Escherichia coli* strain containing a plasmid‐based heterologous MVA pathway^15^ and a refactored GPP synthase and variable mTC/S on a separate plasmid for easy switching and mutagenesis.^13^ Expression of wild‐type bCinS in this platform resulted in high cineole titres (479 mg L_org_^−1^) with only minor amounts of by‐products (2 % α‐terpineol, <1 % camphene, <1 % β‐pinene, and <1 % limonene). Moreover, <2 % of all by‐products do not originate from the α‐terpinyl cation and subsequent water attack step. This is in contrast to CinS_Sf, which accumulates significant amounts of alternative products, including α‐terpineol (6 %) as well as β‐pinene (9 %), α‐pinene (4 %), β‐myrcene (4 %), and sabinene (3 %), with the latter products all originating from branching and/or premature quenching prior to water attack of the α‐terpinyl cation. See Table S3 in the Supporting Information online for a full breakdown of the product profiles. It has been demonstrated that other plant CinSs also accumulate alternative products at significant amounts when expressed in the monoterpenoid production platform. For example, CinS from *A. thaliana* produces only 42 % cineole with α‐terpineol (19 %), β‐myrcene (16 %), and sabinene (14 %) making up the majority of the rest of the product profile.^13^, ^16^ The question arises, how is bCinS able to tightly control the carbocation intermediates without leakage to other products and/or premature quenching? Also, what (stereochemical) intermediates are formed during the cyclisation cascade? We focused our attention on the water attack step in the cyclisation cascade, leading to the α‐terpineol intermediate, as this is the crucial step in cineole formation, in effect “blocking” formation of non‐hydroxylated products from the α‐terpinyl cation.

**Figure 2:**
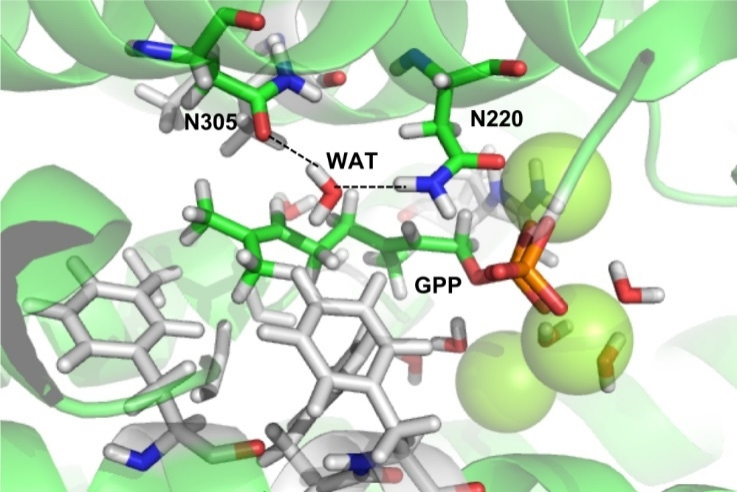
The active site of wild‐type bCinS showing GPP (green carbon atoms) and a water molecule important for cineole formation coordinated by N305 and N220 in a representative structure from cluster analysis of the MD trajectory.

**Figure 3:**
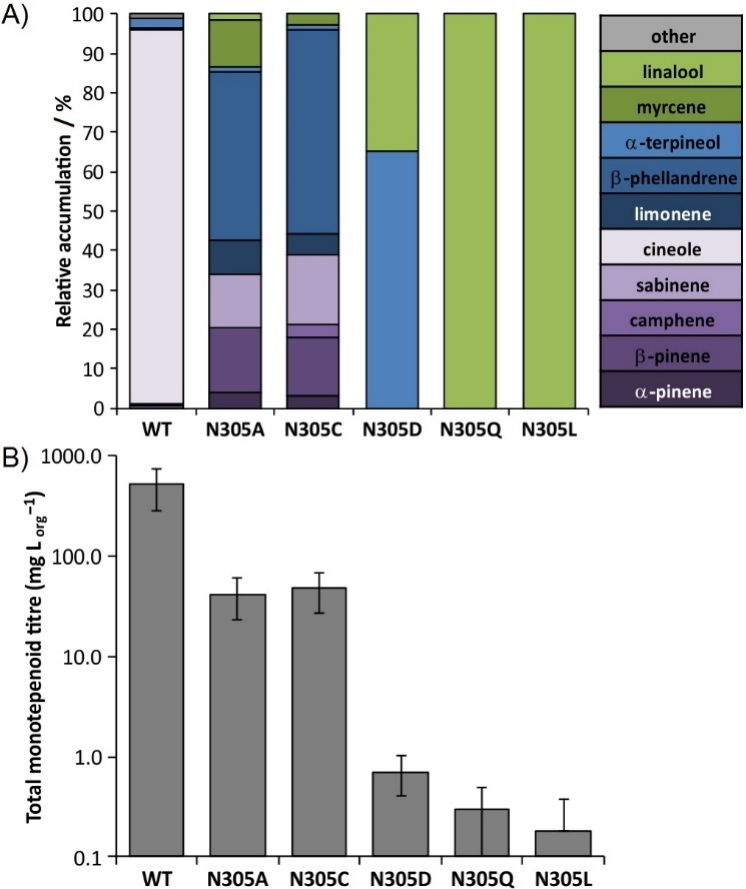
Relative A) product profiles and B) titres achieved upon insertion of the bCinS‐N305 variant enzymes in the *E. coli* monoterpenoid production strain. Bicyclic monoterpenoids are shaded in purple, monocyclic monoterpenoids in blue and linear monoterpenoids in green. Error bars represent the standard deviation of 3–6 biological replicates. Geraniol and derivatives were omitted from the comparison as they are mainly produced by endogenous *E. coli* activity.^13^, ^17^ A full breakdown of the product profiles can be found in Table S3.

From the crystal structure of bCinS, Asn305 was identified as being likely important for water attack, as it is involved in coordination of a water molecule together with Asn220 (Figure 2).^7^ We started by making a series of Asn305 variants, and determined if the mutations had any effect on the product outcome. The N305A variant shows that Asn305 is essential for the reaction with water and cineole formation: the variant does not produce detectable amounts of cineole and mostly produces monoterpene hydrocarbons redirected from the α‐terpinyl cation (including β‐phellandrene, β‐pinene, and sabinene), with only very small amounts of α‐terpineol (<1 %) produced. None of the other mutants (Cys, Asp, Gln or Leu) were able to restore α‐terpineol or cineole formation, suggesting that the unique geometry of Asn is essential for the tight control of the water attack step in bCinS. Full product profiles are shown in Figure 3 and Table S3. Interestingly, only the N305A and N305C variants resulted in reasonably active enzymes; the N305D, Q and L variants each produced monoterpenoid titres of <1 mg L_org_^−1^, which suggests that bCinS does not show a high degree of functional plasticity, unlike plant mTC/S, including CinS_Sf, where Asn338 can be replaced by many other residues resulting in active variants with alternative product profiles.12b, 12c

**Figure 4:**
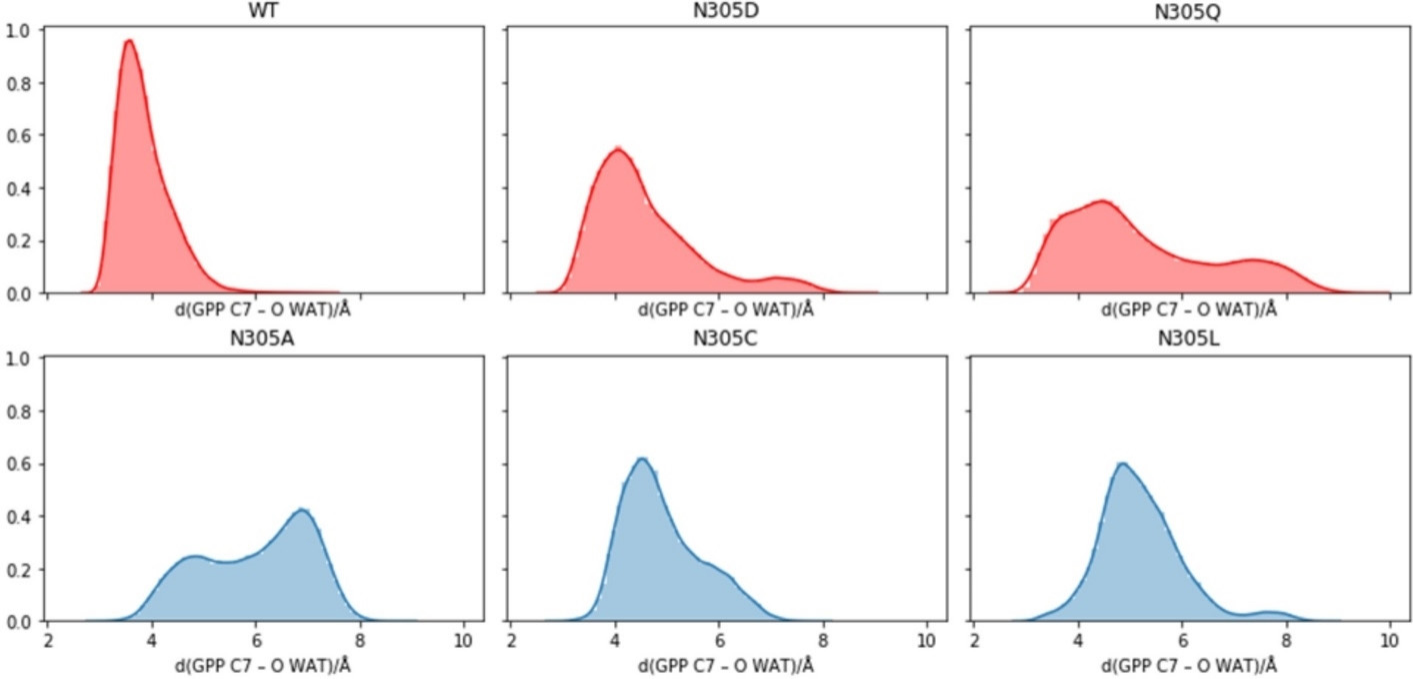
Histograms of the distance between C7 of GPP and O of the closest water in MD simulations of wild‐type bCinS and N305D, Q, A, C and L mutants. The histograms are based on the data from three independent 100 ns MD simulations performed for each model (data from the first 20 ns of each trajectory is considered equilibration and not included here).

To further investigate the role of Asn305 in the interaction of bCinS with water, we performed molecular dynamics (MD) simulations of the ternary geranyl‐PP complexes of wild‐type bCinS and the five 305 variants. Three independent 100 ns simulations were performed for each variant, starting from the structure with a fluorinated analogue (PDB ID: https://www.rcsb.org/structure/5NX7).^7^ The tight hydrogen bond between Asn305 and the water molecule remains intact during simulations of the wild‐type bCinS GPP complex. The water molecule does not stay in position in any of the Asn305 mutants simulated (Ala, Cys, Leu, Gln, and Asp) however, which agrees with the observed product profiles for these variants. Histograms of the distance between C7 and the O atom of the closest water molecule show a strong peak at ≈4 Å for wild‐type bCinS, with this peak broadening in the N305D and N305Q simulations (Figure 4), but the water molecule closest to C7 is not always coordinated by Asp or Gln so it may not be “activated” for reaction.

In the N305A, N305C and N305L variants the histograms show a broad distribution of distances, peaking at a much longer separation. A disrupted water network in all variants is also observed when looking at the heavy atom separation between the donor/acceptor of the residue 305 side chain and the oxygen atom of the closest water molecule during the simulation (Figure S4). There are sharp peaks at ≈2.5 Å for wild‐type bCinS and N305D but the distribution is very broad for the N305Q variant. The distribution of distances for Asn220 ND2 and the closest water molecule has a single sharp peak at ≈3 Å for wild‐type bCinS, but two much broader peaks in all the variants. However, there are still some configurations capable of forming hydrogen bonds between Asn220 and a water molecule in all variants.

This much less disrupted interaction with water during simulations of the Asn305 variants indicates that Asn220 itself is likely not directly involved in water activation (Figure S5). The above results demonstrate the critical importance of Asn305 in coordinating the reaction of the α‐terpinyl cation with water leading to the α‐terpineol intermediate. In order to form cineole, the double bond of the α‐terpineol intermediate needs to be protonated followed by a second cyclisation of the hydroxy group and C7 of α‐terpineol. Our high‐level (SCS‐MP2/6–311+G(d,p)) model calculations show that the water attack on the α‐terpinyl cation to form a hydronium ion (R‐OH_2_^+^) is highly favourable (Figure S7). Protonation of the double bond by internal proton transfer is uphill, but the overall reaction will be driven by the very facile, highly exothermic ring closure of the resulting α‐terpineol cation (note that in the enzyme, the internal proton transfer pushes the positive charge of the cation towards to the phosphate so this step is therefore likely less uphill than in this simple model). Protonation of the other carbon of the double bond is highly unfavourable, as this would lead to a secondary, rather than tertiary, carbocation, and in our model calculations this spontaneously rearranges to the tertiary cation. The proton acceptor for the final step is unknown, and there is no obvious proton relay network present like the one proposed for CinS from *Nicotiana forgetiana*, where the hydroxy group of Tyr496 or Thr278 could act as proton acceptor for deprotonation of (*R*)‐(+)‐α‐terpineol or (*S*)‐(−)‐α‐terpineol, respectively.^16^ The most likely candidate for proton abstraction in bCinS is Asn305, due to its close proximity to C7 and strong interaction with the water molecule during simulation. Asn is not a typical proton acceptor, but nevertheless, our high‐level QM calculations suggest that proton transfer to the amide oxygen is favourable in this case.

**Figure 5:**
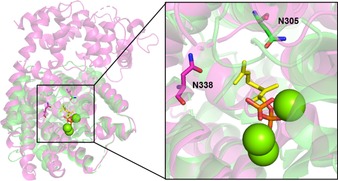
Structural overlay of bacterial CinS from *S. clavuligerus* (bCinS; PDB ID: https://www.rcsb.org/structure/5NX7)^6^ (green) with a plant CinS from *S. fruticosa* (CinS_Sf; PDB ID: https://www.rcsb.org/structure/2J5C)^10^ (purple). The fluorinated substrate analogue and Mg^2+^ ions, as bound to bCinS, are shown in yellow sticks and green spheres, respectively. Asn305 in bCinS and Asn338 in CinS_Sf are indicated and shown as sticks.

A structural comparison of bCinS with CinS from *S. fruticosa* (CinS_Sf) reveals some interesting features; Asn338 in CinS_Sf is located on the opposite side of the active site to Asn305 in bCinS (Figure 5). The location of these Asn residues with presumed similar functions on opposite sides could point to a mechanism involving opposite stereo‐chemical intermediates in bCinS and CinS_Sf. Chiral GC analysis of the α‐terpineol intermediates/by‐products produced by bCinS and CinS_Sf revealed that, although both bCinS and CinS_Sf accumulate both isomers, the (*R*)‐(+)‐isomer is preferentially accumulated by CinS_Sf (64 % of total α‐terpineol formation, with an *R*:*S* ratio of 1.8:1), similar to the related enzyme from *Salvia officinalis*,14b and the (*S*)‐(−)‐isomer is preferentially accumulated by bCinS (91 % of total α‐terpineol formation, with an *S*:*R* ratio of 9.9:1). See Figure S3 and Table S4 for full analysis. This is in agreement with the predicted formation of the (*S*)‐(−)‐α‐terpineol intermediate in simulations involving bCinS (See Experimental Section and Figure S6) and a previous isotope labelling study confirming (*S*)‐α‐terpinyl as intermediate.^18^ So not only is bCinS able to tightly control the Asn305 assisted water attack of the α‐terpinyl cation, it is also able to control the formation and stabilisation of a single linalyl diphosphate isomer intermediate in the early stages of the cyclisation cascade, ultimately leading to almost pure cineole formation.

## Conclusion

In summary, our results show that Asn305 is of critical importance for water activation in bCinS, and mutation of Asn305 results in variants that accumulate products that are re‐directed from the α‐terpinyl cation due to a disrupted water network lacking a coordinated water molecule. Even though Asn was previously implicated in water attack in plant CinS, Asn305 is located on the opposite side of the active site in bCinS, and in addition appears to be involved in the final stages of the cyclisation cascade in an for Asn unusual role as (transient) proton acceptor. Unlike CinS_Sf, many of the bCinS N305 mutants were barely active, demonstrating that bCinS does not show the same level of functional plasticity as observed for many plant mTC/Ss. Unlike some plant CinSs, bCinS also shows a high preference for the formation of the (*S*)‐(−)‐α‐terpinyl cation. This tight control of carbocation formation and water attack coordinated by Asn305 in bCinS results in a high‐fidelity enzyme that is capable of producing cineole to high purity. Our inter‐disciplinary experimental‐computational approach gives important insight into the reaction mechanism of these complex terpene synthase enzymes. This deep understanding of the role of the enzyme in terpene cyclisation synthesis will guide rational engineering efforts towards the predictable tuning of terpene synthase activity for efficient production of desired terpenoids.

## Experimental Section

Generation of the various mutants, in vivo monoterpenoid production conditions, product analysis, MD simulation and QM calculation methods are described in the Supporting Information.

## Conflict of interest

*The authors declare no conflict of interest*.

## Supporting information

As a service to our authors and readers, this journal provides supporting information supplied by the authors. Such materials are peer reviewed and may be re‐organized for online delivery, but are not copy‐edited or typeset. Technical support issues arising from supporting information (other than missing files) should be addressed to the authors.

Supplementary
