# Supplementary material for: Taming the Reactivity of Monoterpene Synthases To Guide Regioselective Product Hydroxylation
Source: Chembiochem. 2019 Dec 3;21(7):985–90. doi: 10.1002/cbic.201900672 (PMC7187147; doi:10.1002/cbic.201900672)
Supplement: Supplementary file 1 — Supplementary [file CBIC-21-985-s001.pdf]

CHEMBIOCHEM

Supporting Information

Taming the Reactivity of Monoterpene Synthases To Guide Regioselective Product Hydroxylation

Nicole G. H. Leferink,[a] Kara E. Ranaghan,[b] Jaime Battye,[a] Linus O. Johannissen,[a] Sam Hay,[a] Marc W. van der Kamp,[b, c] Adrian J. Mulholland,[b] and Nigel S. Scrutton*[a]

cbic_201900672_sm_miscellaneous_information.pdf

Table of Contents

EXPERIMENTAL SECTION 3
Chemicals 3
Bacterial strains and media 3
Site-directed mutagenesis 3
Table S1: Oligonucleotides used for site-directed mutagenesis. 3
Monoterpenoid production in *E. coli* 3
Table S2: Plasmids used in this study 4
GC-MS analysis 4
GC analysis 4
Simulations of the ternary complexes of wt- and mutant-bCinS with 3 Mg^{2+} ions and GPP 5
Ring-closure and proton transfer calculations 5
SUPPLEMENTARY RESULTS 7
GC and GCMS analysis of monoterpenoids produced by CinS 7
Figure S1: GCMS analysis of monoterpenoid production strains containing wt-bCinS and CinS_Sf. 7
Figure S2: GCMS analysis of monoterpenoid production strains containing wt-bCinS and bCinS-N305 mutants. .8
Table S3: Full product profiles and titres obtained for CinS_Sf, wt-bCinS and N305 variants. 9
Figure S3: Chiral GC analysis of α-terpineol produced by bCinS and CinS_Sf. 10
Table S4: Relative accumulation of α-terpineol isomers by wt-bCinS and CinS_Sf. 10
MD results 11
Figure S4: Histograms of the hydrogen bond donor to acceptor distance for the sidechain of residue 305 with the closest water molecule. 11
Figure S5: Histograms of the distance between N220 ND2 and O of the closest water in MD simulations of wt-bCinS and N305D, Q, A, C and L mutants. 12
Figure S6: QM/MM umbrella sampling simulations of the ionisation step in the reaction of NPP in bCinS at the B3LYP/def2-SVP/CHARMM27 level of theory. 13
REFERENCES 14

EXPERIMENTAL SECTION

Chemicals

All monoterpenoid standards used in this study, including α-pinene, β-pinene, camphene, sabinene, β-myrcene, limonene, linalool, α-terpineol and geraniol, were obtained from Sigma-Aldrich with the exception of 1,8-cineole, which was obtained from Tokyo Chemical Industry (TCI).

Bacterial strains and media

All *E. coli* strains were routinely grown in Lysogeny Broth (LB, Formedium) or on LB agar plates including antibiotic supplements as appropriate (carbenicillin, 100 µg mL^{-1}; kanamycin, 50 µg mL^{-1}). Site-directed mutagenesis and plasmid propagation was performed using *E. coli* Stellar cells (Clontech). Monoterpenoid production was performed in phosphate buffered Terrific Broth (TB, Formedium) using *E. coli* DH5α cells (NEB 5α, New England Biolabs).

Site-directed mutagenesis

Mutations were introduced in bCinS using the QuikChange site-directed mutagenesis method (Stratagene) according to the manufacturer's instructions, using plasmid pGPPSmTC/S39 [1] encoding native bCinS as template. The oligonucleotides used are shown in Table S1. Correct introduction of mutations was confirmed by standard Sanger Sequencing (Eurofins).

Table S1: Oligonucleotides used for site-directed mutagenesis. Changed codons are underlined and changed nucleotides are in lower case. Forward oligonucleotides shown only.

Mutation	Primer name	Sequence (5' → 3')
N305A	bCinS_N305A_Fw	CGTGCAGCCATTCGTGGT<u>gcg</u>TATGATTGGTGTGCCGAAAC
N305D	bCinS_N305D_Fw	CGTGCAGCCATTCGTGGT<u>gAT</u>TATGATTGGTGTGCCGAAAC
N305C	bCinS_N305C_Fw	CGTGCAGCCATTCGTGGT<u>tgT</u>TATGATTGGTGTGCCGAAAC
N305Q	bCinS_N305Q_Fw	CGTGCAGCCATTCGTGGT<u>cag</u>TATGATTGGTGTGCCGAAAC
N305L	bCinS_N305L_Fw	CGTGCAGCCATTCGTGGT<u>ctg</u>TATGATTGGTGTGCCGAAAC

Monoterpenoid production in *E. coli*

For monoterpenoid production a pGPPSmTC/S plasmid (Table S2) harbouring native or a variant bCinS gene was co-transformed with pMVA (encoding a heterologous MVA pathway, see Table S2) into *E. coli* DH5α and grown as described previously [2]. Freshly transformed colonies were used to inoculate 3 ml TB supplemented with 0.4 % (w/v) glucose and the appropriate antibiotics in 28 ml glass screw capped vials (Samco) and incubated at 37°C (200 rpm) for approximately 7 hours until growth was apparent. Expression was induced by the addition of 50 µM (isopropyl β-D-1-thiogalactopyranoside) IPTG and 25 nM anhydro-tetracycline (aTet) and incubation was continued for 48 hours at 30°C (200 rpm). A 20% n-nonane (v/v) organic layer was added to capture the volatile terpenoid products at the time of induction. After induction, the nonane overlay was collected, dried over anhydrous $MgSO_4$ and mixed at a 1:1 ratio with ethyl acetate containing 0.01% (v/v) *sec*-butylbenzene as internal standard for GC or GC-MS analysis.

Table S2: Plasmids used in this study

Plasmid reference	Plasmid name	Description	Source
pMVA	pBbA5a-MTSAe-T1f-MBI(f)-T1002i	p15A, Kanr, PlacUV5, MTSA, T1, MBI-f, T1002	[2]
pGPPSmTC/S5	pBbB2a-trAgGPPS(co)-trCinS_Sf	pBBR, Ampr, Ptet, trAgGPPS(co)- trCinS_Sf	[2]
pGPPSmTC/S39	pBbB2a-trAgGPPS(co)- bCinS	pBBR, Ampr, Ptet, trAgGPPS(co)- bCinS	[1]
pGPPSmTC/S39-N305A	pBbB2a-trAgGPPS(co)- bCinS-N305A	pBBR, Ampr, Ptet, trAgGPPS(co)- bCinS-N305A	This study
pGPPSmTC/S39-N305D	pBbB2a-trAgGPPS(co)- bCinS-N305D	pBBR, Ampr, Ptet, trAgGPPS(co)- bCinS-N305D	This study
pGPPSmTC/S39-N305C	pBbB2a-trAgGPPS(co)- bCinS-N305C	pBBR, Ampr, Ptet, trAgGPPS(co)- bCinS-N305C	This study
pGPPSmTC/S39-N305Q	pBbB2a-trAgGPPS(co)- bCinS-N305Q	pBBR, Ampr, Ptet, trAgGPPS(co)- bCinS-N305Q	This study
pGPPSmTC/S39-N305L	pBbB2a-trAgGPPS(co)- bCinS-N305L	pBBR, Ampr, Ptet, trAgGPPS(co)- bCinS-N305L	This study

GC-MS analysis

The samples were injected onto an Agilent Technologies 7890B GC equipped with an Agilent Technologies 5977A MSD. The products were separated on a DB-WAX column (30 m x 0.32 mm i.d., 0.25 µM film thickness, Agilent Technologies). The injector temperature was set at 240°C with a split ratio of 20:1 (1 µl injection). The carrier gas was helium with a flow rate of 1 ml min^{-1} and a pressure of 5.1 psi. The following oven program was used: 50°C (1 min hold), ramp to 68°C at 5°C min^{-1} (2 min hold), and ramp to 230°C at 25°C min^{-1} (2 min hold). The ion source temperature of the mass spectrometer (MS) was set to 230°C and spectra were recorded from m/z 50 to m/z 250. Compound identification was carried out using authentic standards and comparison to reference spectra in the NIST library of MS spectra and fragmentation patterns as described previously [2]. Monoterpenoids were quantified using authentic standards wherever possible using experimentally determined relative response factors in relation to the internal standard used. In the absence of an authentic standard, concentrations were estimated using a relative response factor of 1.

GC analysis

To determine the chirality of the α-terpineol intermediate produced by bCinS, samples were analysed by gas-chromatography on an Agilent Technologies 7890A GC system equipped with an FID detector, a 7693 autosampler, and a CP-Chirasil-DEX-CB column (25 m × 0.25 mm i.d., 0.25 µm film thickness, Agilent Technologies). The nonane overlays and authentic (+)- and (-)-α-terpineol standards were analysed using the following method: the injector temperature was at 180°C, and 1 µL of sample was injected split-less. The carrier gas was helium with a flow rate of 1 mL/min and a pressure of 11.3 psi. The oven program began at a temperature of 70°C which was then increased to 90°C at 8°C/min. This was followed by an increase in temperature to 150°C at a rate of 2°C/min and then to 190°C at 40°C/min (1 min hold). The FID detector was maintained at a temperature of 200°C with a flow of hydrogen at 30 mL/min.

Simulations of the ternary complexes of wt- and mutant-bCinS with 3 Mg^{2+} ions and GPP

The structure of bCinS reported in our previous work was used to build the models simulated here [1]. Asn305 was mutated to Ala, Cys, Asp, Leu and Gln using the Mutagenesis wizard in Pymol and the same protocol was then followed for the wt and mutant models. The protonation states of titratable residues were estimated using PropKa3.1 [3] and the enzyme was solvated using a box of TIP3P [4] water molecules (with a minimum buffer of 13 Å around the protein) using the solvate plugin of the VMD package [5]. Counter-ions were added to neutralize the system using autoionize plugin of VMD [5]. The CHARMM27 forcefield [6] was used to describe the protein with parameters for GPP were adapted from those used for FPP in the work of van der Kamp *et al*.[7] The parameter set developed by Allner *et al*. [8] was used to describe the three Mg^{2+} ions. The setup of the model consisted of: (i) minimization of the positions of the hydrogen atoms (all heavy atoms fixed); (ii) minimization of the solvent (with all protein heavy atoms fixed); (iii) energy minimization of the entire system with positional restraints of 5 kcal mol^{-1} $Å^{-2}$ applied to all Cα atoms; (iv) canonical ensemble (NVT) thermalisation to1 300 K over 20 ps with positional restraints of 5 kcal mol^{-1} $Å^{-2}$ on Cα atoms; (v) thermal equilibration at 300K for 100 ps with positional restraints of 5 kcal mol^{-1} $Å^{-2}$ on Cα atoms; (vi) 140 ps of NPT equilibration with gradually decreasing restraints on the Cα atoms; (vi) 100 ns production simulation. Three sets of isothermal-isobaric ensemble (NPT) MD simulations were performed at 300 K for each enzyme variant, repeating steps (iv)-(vi) to obtain 3 models with different initial conditions. MD simulations were carried out on GPUs using the PMEMD code [9] of AMBER16 [10]. Langevin dynamics was used for temperature control (collision frequency of 5 ps^{-1} for equilibration and 2 ps^{-1} for production), and pressure was controlled by coupling to an external bath (AMBER16 default settings) for NPT conditions. Average linkage hierarchical clustering (after alignment of structures based on positions of active site residues) was carried out using the CPPTRAJ utility of AMBERTOOLS16 [10] to identify representative structures of the ternary complex over the course of the simulations. Hydrogen bond analysis was carried out using the CPPTRAJ utility of AMBERTOOLS16 [10] with Asn220 as a hydrogen bond donor and water as hydrogen bond acceptor and residue 305 as the hydrogen bond donor and water as a hydrogen bond acceptor to compare with wt-bCinS, where a water molecule is held in place by these 2 residues, see Figure 2.

α-Terpinyl cation formation

A model of bCinS with neryl diphosphate (NPP) was prepared in the same way as described for GPP above. QM/MM MD simulations at the B3LYP/def2-SVP/CHARMM27 level of theory were carried out using the ORCA interface with AMBER16. NPP and the 3 Mg^{2+} ions were treated by QM with the rest of the model treated by MM with the CHARMM27 forcefield. In an attempt to model the ionisation step of the mechanism, the P—O distance in NPP was used as a reaction coordinate, increasing the distance from 1.5 Å in 0.1 intervals. Simulations were carried out using a harmonic restraint of force constant of k = 200 kcal/mol $Å^{-2}$ applied to the reaction coordinate with 2 ps sampling carried out at each value of the reaction coordinate. Visual inspection of structures along the reaction coordinate showed that at a value of *d*(P—O) = 2.2 Å on the reaction coordinate the cation had spontaneously cyclised to form the (-)-α-terpinyl cation, see Figure S7 below.

Ring-closure and proton transfer calculations

Quantum mechanical calculations were performed on each intermediate from the α-terpinyl cation to the cineole product, in Gaussian 09 revision D.01, [11] using Spin-Component Scaled Second-Order Møller−Plesset Perturbation Theory [12] (MP2 [13] with IOp(3/125=0333312000) in the G09 route card) and

6-311+G(d,p), *i.e.* the 6-311G basis sets [14] with diffuse functions and d and p polarization functions. Energy minimisations were carried out in implicit solvation using the polarisable continuum model (PCM) to represent water (ε = 78) as well as a generic solvent with a dielectric constant ε = 4. The Counterpoise method [15] was used to account for the basis set superposition error (BSSE) in the water attack step (**1** $\rightarrow$ **2** in Figure S7); for the final proton transfer step (**4** $\rightarrow$ **5** in Figure S7) the proton donor and acceptor molecules were calculated separately, and since only a proton is added / removed no BSSE correction is required. Transition states for the cyclisation and internal proton transfer steps were also optimised, and frequency calculations confirmed that each has only one imaginary frequency.

SUPPLEMENTARY RESULTS

GC and GCMS analysis of monoterpenoids produced by CinS

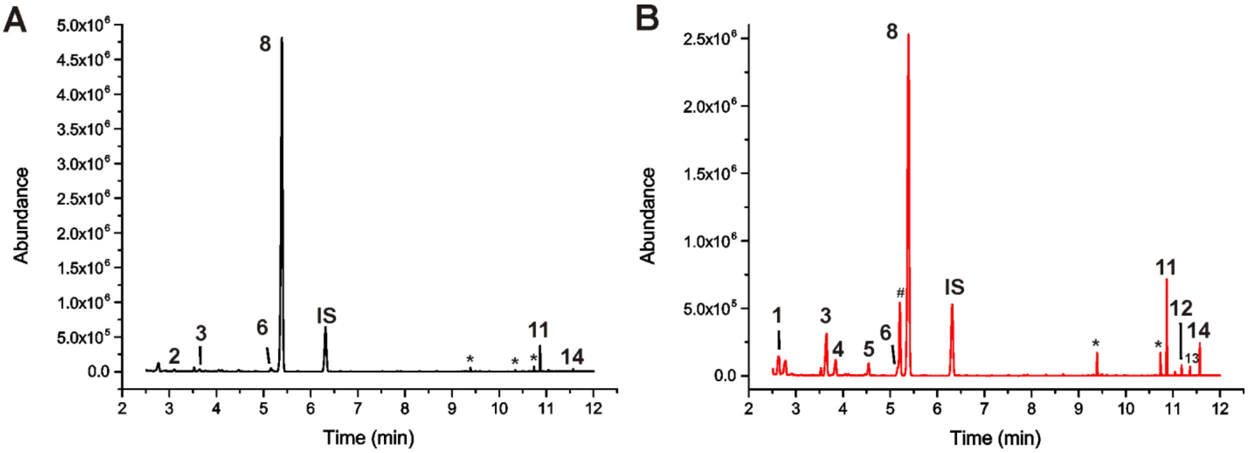

Figure S1: GCMS analysis of monoterpenoid production strains containing wt-bCinS and CinS_Sf. Representative total ion count chromatograms of CinS from *Streptomyces clavuligerus* (bCinS) (**A**) and CinS from *Salvia fruticosa* (CinS_Sf) (**B**). Detected monoterpenoid peaks are: α-pinene (**1**, rt = 2062 min); camphene (**2**, rt = 3.11 min); β-pinene (**3**, rt = 3.65 min); sabinene (**4**, rt = 3.84 min); β-myrcene (**5**, rt = 4.54 min); limonene (**6**, rt = 5.16 min); β-phellandrene (**7**, rt = 5.34 min); 1,8-cineole (**8**, rt = 5.39 min); linalool (**9**, rt = 9.98 min); neral (**10**, rt = 10.78 min); α-terpineol (**11**, rt = 10.89 min); geranial (**12**, rt = 11.04 min); nerol (**13**, rt = 11.36 min); and geraniol (**14**, rt = 11.57 min). IS = internal standard (*sec*-butylbenzene). *Minor amounts of β-, 4-, and δ-terpineol. #Dodecane contamination.

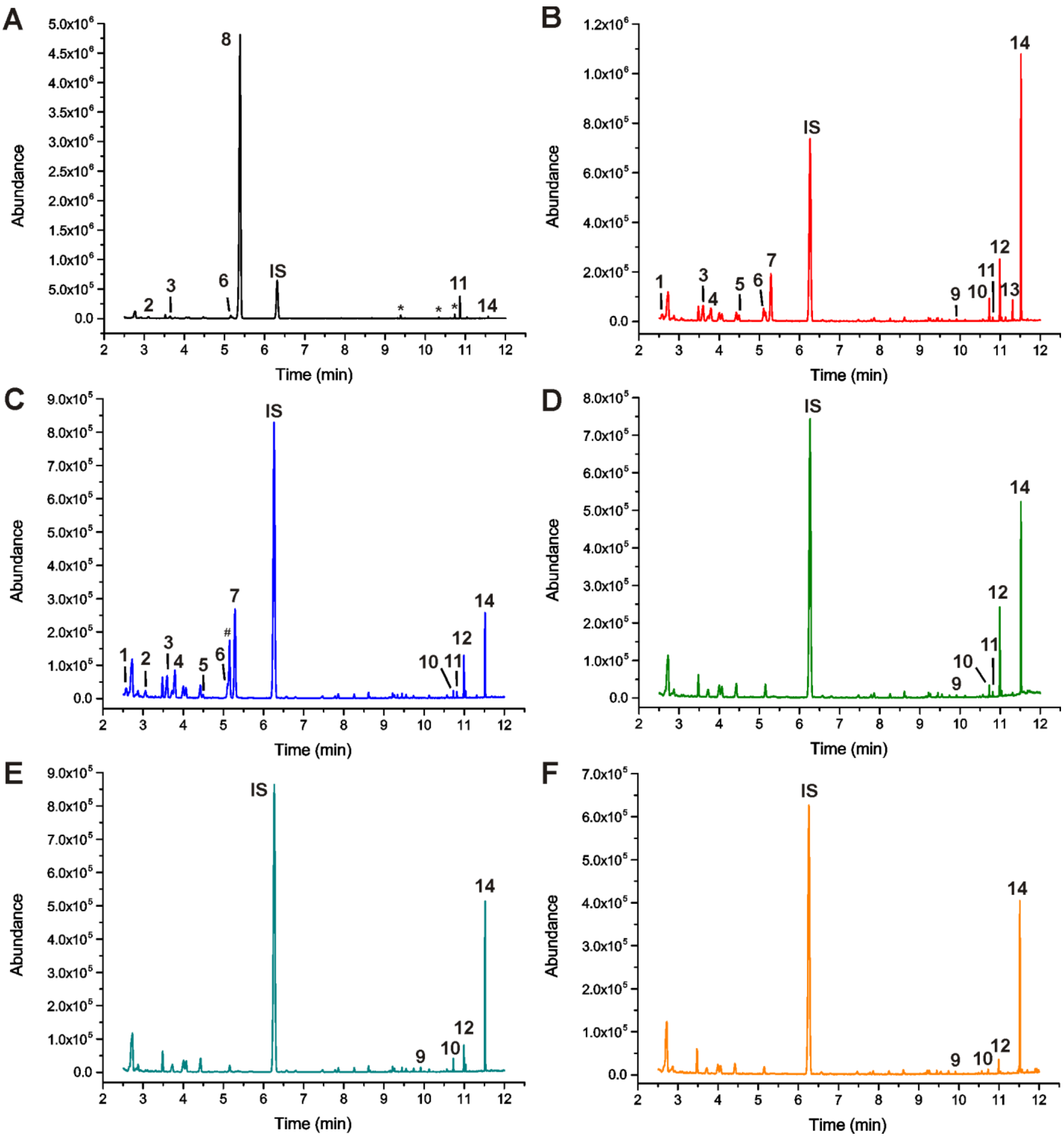

Figure S2: GCMS analysis of monoterpenoid production strains containing wt-bCinS and bCinS-N305 mutants. Representative total ion count chromatograms of wt-bCinS (**A**), bCinS-N305A (**B**), bCinS-N305C (**C**), bCinS-N305D (**D**), bCinS-N305Q, (**E**), and bCinS-N305L (**F**). Detected monoterpenoid peaks are: α-pinene (**1**, rt = 2062 min); camphene (**2**, rt = 3.11 min); β-pinene (**3**, rt = 3.65 min); sabinene (**4**, rt = 3.84 min); β-myrcene (**5**, rt = 4.54 min); limonene (**6**, rt = 5.16 min); β-phellandrene (**7**, rt = 5.34 min); 1,8-cineole (**8**, rt = 5.39 min); linalool (**9**, rt = 9.98 min); neral (**10**, rt = 10.78 min); α-terpineol (**11**, rt = 10.89 min); geranial (**12**, rt = 11.04 min); nerol (**13**, rt = 11.36 min); and geraniol (**14**, rt = 11.57 min). IS = internal standard (*sec*-butylbenzene). *Minor amounts of β-, 4-, and δ-terpineol detected for wt-bCinS. #Dodecane contamination.

Table S3: Full product profiles and titres obtained for CinS_Sf, wt-bCinS and N305 variants. Product profiles and monoterpenoid titres (mg L_{org}^{-1}) were determined from two-phase cultures with a nonane overlay for each *E. coli* strain containing the MVA pathway and a bCinS variant. Averages of 3-5 biological replicates and the corresponding standard deviations are shown. ND = not detected.

Variant	α-pinene	camphene	β-pinene	sabinene	β-myrcene	limonene	β-phellandrene	1,8-cineole	linalool	α-terpineol	geranoids[a]	other[b]
CinS_Sf	19.8 ± 11.3	0.4 ± 0.3	49.3 ± 28.5	16.9 ± 11.0	19.8 ± 11.1	4.5 ± 2.9	ND	358.6 ± 204.6	ND	30.5 ± 17.6	14.4 ± 9.7	14.1 ± 10.3
wt-bCinS	0.6 ± 0.1	2.8 ± 1.4	3.5 ± 1.5	0.4 ± 0.3	0.6 ± 0.6	2.6 ± 1.3	ND	514.5 ± 211.2	ND	11.6 ± 4.9	2.7 ± 2.1	3.1 ± 1.4
bCS-N305A	1.8 ± 0.6	ND	7.1 ± 2.8	5.8 ± 2.3	5.1 ± 2.6	3.8 ± 1.6	18.5 ± 7.7	ND	0.7 ± 0.3	0.5 ± 0.1	57.1 ± 19.7	ND
bCS-N305C	1.5 ± 1.4	1.5 ± 1.4	7.2 ± 2.6	8.6 ± 3.1	1.3 ± 0.7	2.6 ± 0.9	25.2 ± 9.8	ND	ND	0.5 ± 0.2	14.5 ± 5.8	ND
bCS-N305D	ND	ND	ND	ND	ND	ND	ND	ND	0.2 ± 0.1	0.5 ± 0.2	45.9 ± 11.1	ND
bCS-N305Q	ND	ND	ND	ND	ND	ND	ND	ND	0.3 ± 0.2	ND	23.3 ± 12.7	ND
bCS-N305L	ND	ND	ND	ND	ND	ND	ND	ND	0.2 ± 0.2	ND	24.3 ± 12.2	ND

[a]Geraniol and derivatives (geranoids) production is most likely the result of endogenous *E. coli* activity, and includes the compounds nerol, neral, and geranial [2, 16]

[b]Other monoterpenoids detected include: β-terpineol, 4-terpineol, and δ-terpineol

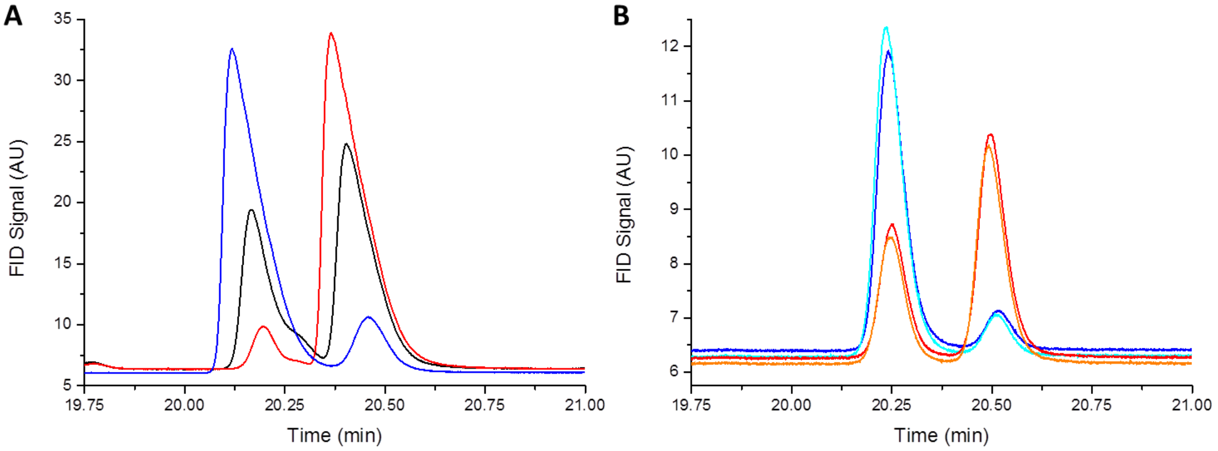

Figure S3: Chiral GC analysis of α-terpineol produced by bCinS and CinS_Sf. A) Separation of commercially available α-terpineol standards: (-)-α-terpineol (blue), (+)-α-terpineol (red), and a mixture of isomers (black). **B**) Separation of α-terpineol isomers produced by engineered *E. coli* cultures containing wt-bCinS (cyan and blue) and CinS_Sf (red and orange). The α-terpineol concentration in the standards is 100 mg L_{org}^{-1}, and the α-terpineol concentration in the samples is 14-15 mg L_{org}^{-1} (mixture of isomers).

Table S4: Relative accumulation of α-terpineol isomers by wt-bCinS and CinS_Sf. The α-terpineol isomers were determined from commercially available α-terpineol standards (0.1-1.0 mg/ml in nonane) and two-phase cultures with a nonane overlay for *E. coli* strains containing the MVA pathway and wt-bCinS or CinS_Sf. Results of 2 biological replicates are shown.

**Sample**	**Relative presence (%)**		**Ratio**
	**(*S*)-(-)-α-terpineol**	**(*R*)-(+)-α-terpineol**	
α-terpineol standard mixture	38.5 ± 0.8	61.5 ± 0.8	1.6:1 (*R*:*S*)
(+)-α-terpineol standard	6.8 ± 0.3	93.2 ± 0.3	13.7:1 (*R*:*S*)
(-)-α-terpineol standard	92.8 ± 6.0	7.2 ± 6.0	12.9:1 (*S*:*R*)
CinS_Sf	36.1 ± 0.3	63.9 ± 0.3	1.8:1 (*R*:*S*)
bCinS	90.8 ± 0.3	9.2 ± 0.3	9.7:1 (*S*:*R*)

MD results

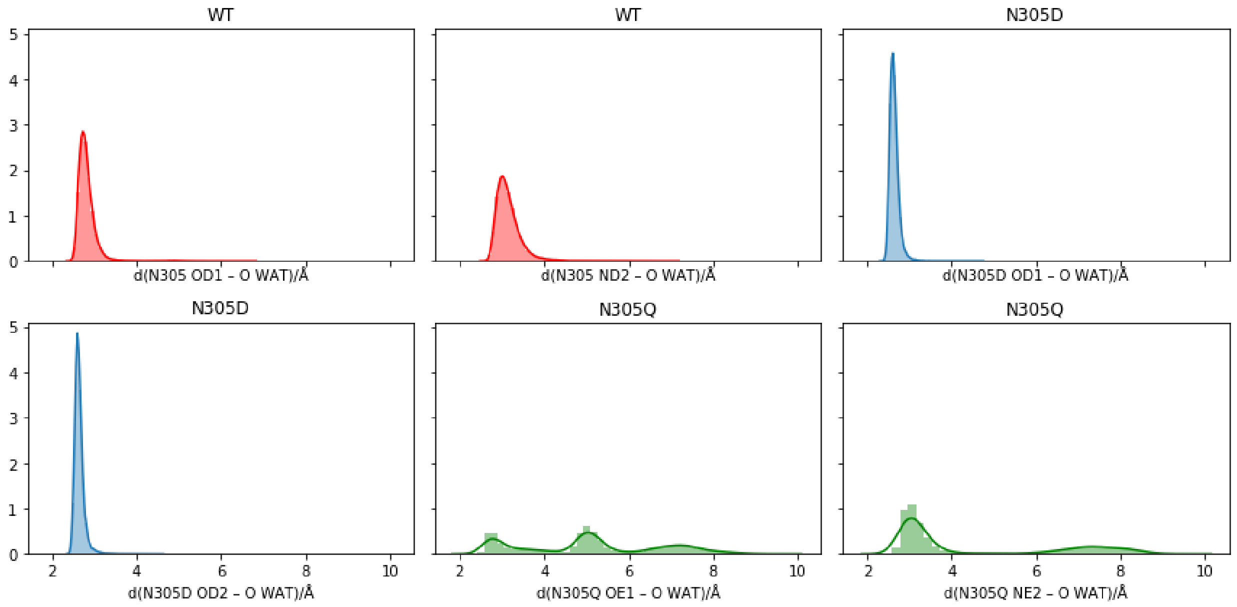

Figure S4: Histograms of the hydrogen bond donor to acceptor distance for the sidechain of residue 305 with the closest water molecule. The histograms are based on the data from the 3 independent 100 ns simulations of wt-bCinS (red) and N305D (blue) and N305Q (green) variants with GPP as a substrate. The first 20 ns were considered equilibration and not considered in the analysis. In wt-bCinS Asn305 and Asn220 coordinate a water molecule important for cineole formation as shown in Figure 2.

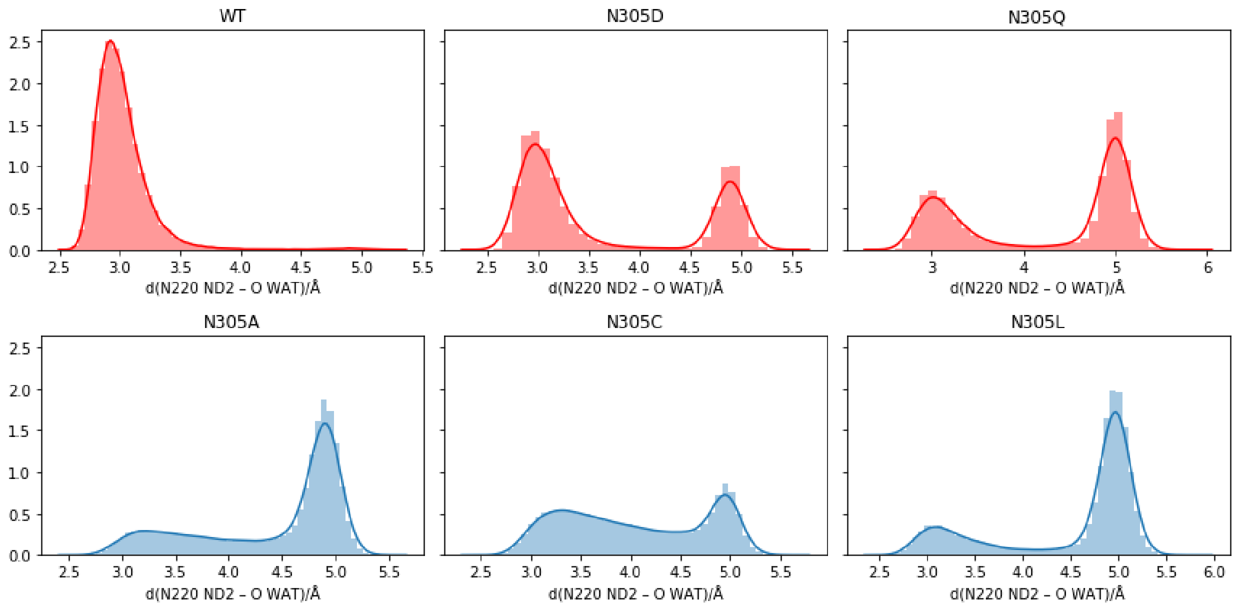

Figure S5: Histograms of the distance between N220 ND2 and O of the closest water in MD simulations of wt-bCinS and N305D, Q, A, C and L mutants. The histograms are based on the data from the 3 independent 100 ns MD simulations performed for each model (data from the first 20 ns of each trajectory is considered equilibration and not included here). For a hydrogen bond to be present between N220 and a water molecule, this heavy atom separation must be less than 3.5 Å. In wt-bCinS, Asn305 and Asn220 coordinate a water molecule important for cineole formation as shown in Figure 2.

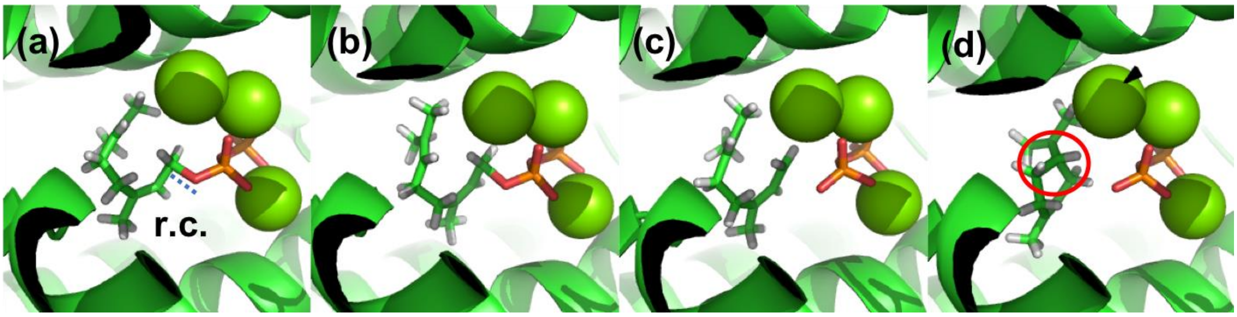

Figure S6: QM/MM umbrella sampling simulations of the ionisation step in the reaction of NPP in bCinS at the B3LYP/def2-SVP/CHARMM27 level of theory. (a) NPP indicating the bond length used as a reaction coordinate (r.c.) (b) r.c. = 1.8 Å (c) r.c. = 2.1 Å (d) r.c. = 2.2 Å where the red circle highlights the newly formed C-C bond in the cyclisation to form the (-)-α-terpinyl cation.

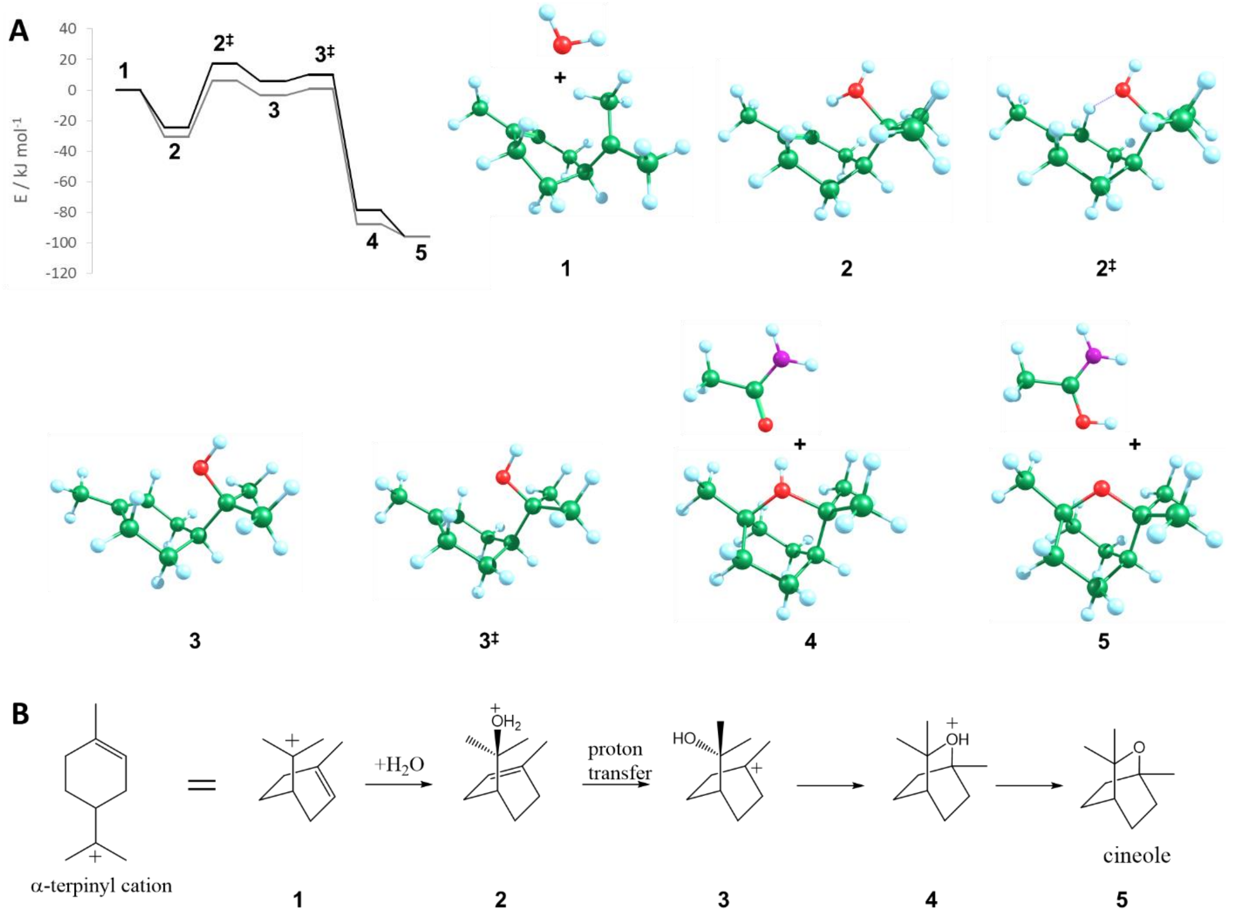

Figure S7: Modelling the formation of cineole from the α-terpinyl cation. (**A**) Potential energy profile and structures for reaction intermediates. Energies for water (black line) and ε=4 (grey lines) solvated models are shown relative to the α-terpinyl cation, with the energy minimised water solvated structures shown (transition states are labelled ‡). The energies of **4** and **5** were normalised by subtracting the energy of the neutral amide. (**B**) Mechanism with intermediates labelled.

REFERENCES

[1] V. Karuppiah, K. E. Ranaghan, N. G. H. Leferink, L. O. Johannissen, M. Shanmugam, A. Ni Cheallaigh, N. Bennett, L. Kearsey, E. Takano, J. Gardiner, M. W. Van der Kamp, S. Hay, A. J. Mulholland, D. Leys, N. S. Scrutton, *ACS Catal.* **2017**, *7*, 6268-6282.

[2] N. G. H. Leferink, A. J. Jervis, Z. Zebec, H. S. Toogood, S. Hay, E. Takano, N. S. Scrutton, *ChemistrySelect* **2016**, *1*, 1893-1896.

[3] a) M. H. M. Olsson, C. R. Søndergaard, M. Rostkowski, J. H. Jensen, *J. Chem. Theory Comput.* **2011**, *7*, 525-537; b) C. R. Søndergaard, M. H. M. Olsson, M. Rostkowski, J. H. Jensen, *J. Chem. Theory Comput.* **2011**, *7*, 2284-2295.

[4] W. L. Jorgensen, J. Chandrasekhar, J. D. Madura, R. W. Impey, M. L. Klein, *J. Chem. Phys.* **1983**, *79*, 926-935.

[5] W. Humphrey, A. Dalke, K. Schulten, *J. Mol. Graphics* **1996**, *14*, 33-38.

[6] A. D. MacKerell, D. Bashford, M. Bellott, R. L. Dunbrack, J. D. Evanseck, M. J. Field, S. Fischer, J. Gao, H. Guo, S. Ha, D. Joseph-McCarthy, L. Kuchnir, K. Kuczera, F. T. K. Lau, C. Mattos, S. Michnick, T. Ngo, D. T. Nguyen, B. Prodhom, W. E. Reiher, B. Roux, M. Schlenkrich, J. C. Smith, R. Stote, J. Straub, M. Watanabe, J. Wiórkiewicz-Kuczera, D. Yin, M. Karplus, *J. Phys. Chem. B* **1998**, *102*, 3586-3616.

[7] M. W. van der Kamp, J. Sirirak, J. Zurek, R. K. Allemann, A. J. Mulholland, *Biochemistry* **2013**, *52*, 8094-8105.

[8] O. Allnér, L. Nilsson, A. Villa, *J. Chem. Theory Comput.* **2012**, *8*, 1493-1502.

[9] A. W. Götz, M. J. Williamson, D. Xu, D. Poole, S. Le Grand, R. C. Walker, *J. Chem. Theory Comput.* **2012**, *8*, 1542-1555.

[10] D. A. Case, R. M. Betz, D. S. Cerutti, T. E. Cheatham, III, T. A. Darden, R. E. Duke, T. J. Giese, H. Gohlke, A. W. Goetz, N. Homeyer, S. Izadi, P. Janowski, J. Kaus, A. Kovalenko, T. S. Lee, S. LeGrand, P. Li, C. Lin, T. Luchko, R. Luo, B. Madej, D. Mermelstein, K. M. Merz, G. Monard, H. Nguyen, H. T. Nguyen, I. Omelyan, A. Onufriev, D. R. Roe, A. Roitberg, C. Sagui, C. L. Simmerling, W. M. Botello-Smith, J. Swails, R. C. Walker, J. Wang, R. M. Wolf, X. Wu, L. Xiao, P. A. Kollman, *AMBER 2016*, University of California, San Francisco, **2016**.

[11] M. J. Frisch, G. Trucks, H. Schlegel, G. Scuseria, M. Robb, J. Cheeseman, G. Scalmani, V. Barone, B. Mennucci, G. Petersson, *Gaussian 09 D. 01*, Wallingford CT **2016**.

[12] M. Gerenkamp, S. Grimme, *Chem. Phys. Lett.* **2004**, *392*, 229-235.

[13] M. J. Frisch, M. Head-Gordon, J. A. Pople, *Chem. Phys. Lett.* **1990**, *166*, 275-280.

[14] A. D. McLean, G. S. Chandler, *J. Chem. Phys.* **1980**, *72*, 5639-5648.

[15] S. Simon, M. Duran, J. J. Dannenberg, *J. Chem. Phys.* **1996**, *105*, 11024-11031.

[16] W. Liu, R. Zhang, N. Tian, X. Xu, Y. Cao, M. Xian, H. Liu, *Bioengineered* **2015**, *6*, 288-293.
